# Heavy-Ion-Induced Lung Tumors: Dose- & LET-Dependence

**DOI:** 10.3390/life12060907

**Published:** 2022-06-17

**Authors:** Polly Y. Chang, James Bakke, Chris J. Rosen, Kathleen A. Bjornstad, Jian-Hua Mao, Eleanor A. Blakely

**Affiliations:** 1 Biosciences Division, SRI International, Menlo Park, CA 94025, USA; polly.chang@sri.com (P.Y.C.); james.bakke@sri.com (J.B.); 2 Biological Systems & Engineering Division, Lawrence Berkeley Laboratory, Berkeley, CA 94720, USA; cjrosen@lbl.gov (C.J.R.); kabjornstad@lbl.gov (K.A.B.); jhmao@lbl.gov (J.-H.M.)

**Keywords:** particle radiation, lung, tumorigenesis, linear energy transfer (LET), low dose

## Abstract

There is a limited published literature reporting dose-dependent data for in vivo tumorigenesis prevalence in different organs of various rodent models after exposure to low, single doses of charged particle beams. The goal of this study is to reduce uncertainties in estimating particle-radiation-induced risk of lung tumorigenesis for manned travel into deep space by improving our understanding of the high-LET-dependent dose-response from exposure to individual ion beams after low particle doses (0.03–0.80 Gy). Female CB6F1 mice were irradiated with low single doses of either oxygen, silicon, titanium, or iron ions at various energies to cover a range of dose-averaged LET values from 0.2–193 keV/µm, using ^137^Cs γ-rays as the reference radiation. Sham-treated controls were included in each individual experiment totally 398 animals across the 5 studies reported. Based on power calculations, between 40–156 mice were included in each of the treatment groups. Tumor prevalence at 16 months after radiation exposure was determined and compared to the age-matched, sham-treated animals. Results indicate that lung tumor prevalence is non-linear as a function of dose with suggestions of threshold doses depending on the LET of the beams. Histopathological evaluations of the tumors showed that the majority of tumors were benign bronchioloalveolar adenomas with occasional carcinomas or lymphosarcomas which may have resulted from metastases from other sites.

## 1. Introduction

The space radiation environment outside the earth’s magnetosphere is complex and includes the possibility of solar particle events (SPE) and galactic cosmic radiation (GCR). Although optimized shielding in space vehicles can protect astronauts against SPEs, highly energetic and charged particle components in GCR can pose significant human health risks, especially in long duration mission to Mars. 

Currently, the U.S. National Aeronautics and Space Administration (NASA) scales cancer incidences or mortality rate estimates from epidemiological data from the effects of exposure to ground-based low-doses and low-dose rates of primarily atomic bomb survivors to different radiation types that are found in space with the Dose and Dose-Rate Effectiveness Factor (DDREF) and radiation quality factors. However, large uncertainties exist in radiation quality factors, dose and dose-rate dependencies, transfer of risk across populations, the determination of space radiation organ exposures and the various errors inherent to human data sources. These uncertainties are related to both quantitative and qualitative differences between high- and low-linear energy transfer (LET) radiations encountered in space travel, and the dose linearity and additivity of effects for different radiation types, especially at low particle fluence with possible synergistic risks from other flight factors with radiation risk [1].

Cancer is a highly heterogeneous pathology with respect to cell type and tissue origin [2]. Cancer is also a disease that can involve deregulation of multiple pathways governing fundamental cell processes such as cell death, proliferation, differentiation and migration [2]. Using animal models for carcinogenesis, there is now evidence that cancer stem cells (CSC) in solid tumors play a role in carcinogenesis [3,4,5,6,7]. There is also evidence that progenitor cells can revert to stem cells upon being damaged [8]. 

Quantitative studies of tissue-specific cancer induction after low particle fluences are important to the understanding of cancer causation by targeted (TE) versus non-targeted (NTE) radiation effects. NASA recognizes the importance of these issues in particle-radiation induced carcinogenesis [9,10] and has several focused radiation programs looking at particle radiation-induced cancers specifically in mammary gland [11]; blood [12]; liver [13] and gastrointestinal systems [14]. 

Several significant findings were reported from studies focused on space-radiation-induced lung tumorigenesis. Results from these studies using different mouse models demonstrated that high-LET radiations resulted in an increased risk of tumorigenesis compared to low-LET radiations. For example, high-LET radiation exposure was shown to accelerate lung cancer progression in the K-ras (LA1) lung cancer mouse model with dose fractionation being more permissive for cancer progression [15]. However, Moding et al., 2016 [16] investigated the role of *p53* in lung carcinogenesis and lymphomagenesis in *LA-1 KrasG12D* mice with *wild-type p53* or an extra copy of *p53* (*super p53*) exposed to fractionated total body irradiation with low linear energy transfer (low-LET) X-rays or high-LET iron ions and compared tumor formation in these mice with unirradiated controls. They found that an additional copy of *p53* suppressed both Kras-driven lung tumor and lymphoma development in the absence of radiation. However, an additional copy of *p53* did not affect lymphoma development following low- or high-LET radiation exposure and was unable to suppress radiation-induced expansion of thymocytes with mutated *Kras*. Moreover, radiation exposure increased lung tumor size in *super p53* but not *wild-type p53* mice. These results demonstrate that although *p53* suppresses the development of spontaneous tumors expressing Kras*G12D*, in the context of exposure to ionizing radiation, an extra copy of *p53* does not protect against radiation-induced lymphoma and may promote Kras*G12D* mutant lung cancer. 

Incidence of lung tumorigenesis in wild-type C57BL/6 mice at 1.5 years after 1 Gy exposure of iron, silicon, oxygen ions or X ray showed that HZE particles induced higher levels of lung tumors when compared to the reference low LET X-rays [17]. In addition, Wang et al. also reported that silicon exposure appears to induce more aggressive lung tumors [17]. To better understand the underlying mechanisms associated with high-LET-induced lung tumorigenesis, lung progenitor cells in the distal airway epithelium were shown to demonstrate increased p53-dependent apoptosis and defects in mitotic progression [18]. 

To date there is no evidence for space radiation-induced cancer in astronauts or cosmonauts [19,20,21,22]. There are good measurements of the types and doses of radiation exposure during transit to and from Mars with either a fly-by or a stay on the surface [23,24,25]. However, no human has yet experienced protracted exposure to the estimated space radiation types, doses and dose rates from a Mars expedition. Quantitative studies of tissue-specific cancer induction after low particle fluences are important to the understanding of cancer causation, (e.g., by radiogenic TE versus NTE) and animal experiments can help bridge this gap. 

The female CB6F1 mouse model has been used to study long-term tumorigenesis for several decades to provide data that can be used for cancer risk analysis for particle radiation exposure [26,27,28,29,30]. Cohorts of animals were irradiated to a broad spectrum of individual particle radiations with various fluences with the primary goal of examining the dose- and LET-dependence of Harderian Gland (HG) tumorigenesis at 16-months after a low single, acute heavy charged particles exposure. In our laboratories, we have continued to use this model and filled in gaps in knowledge by reporting on HG tumor prevalence after additional ions and published new computational models for analysis [31,32]. In this report our focus is to investigate the role of low particle fluence- and LET-dependencies of simulated space radiation-induced lung tumorigenesis using this same animal model to provide insights into the consideration of tissue-specificity in risk analysis. 

## 2. Materials and Methods

### 2.1. Animal Procedures

BALB/cAnNHsd inbred females were crossed with C57BL/6NHsd males to create the CB6F1/Hsd model at Envigo Laboratories (Frederick, MD, USA). Source animal cohorts were maintained in the same barrier facility at the vendor for consistency. Greater than 2500 animals were used over the period of 8 years for this research, which included the time needed to breed the animals and husbandry period of 16-month post irradiation. Sham-treated controls were included in each individual experiment totally 398 animals across the 5 radiation studies reported (see Table 1 and Table 2). Based on power calculations, we included 40–156 mice in the different radiation treatment groups reported in Table 3, Table 4, Table 5, Table 6 and Table 7. For each study, appropriate numbers of female animals (100–120 days old) were microchipped with AVID^®^ chips for identification and were shipped by the animal provider, Envigo in climate-controlled trucks directly to the Brookhaven Lab Animal Facility (BLAF) at Brookhaven National Laboratory (BNL), Upton New York approximately 1 week prior to the assigned beam time. 

Animals were weighed and randomized by weight just prior to whole-body particle radiation exposure. Within 1 week after radiation exposure, animals were shipped to the animal facility at the Lawrence Berkeley National Laboratory (LBNL) and maintained for 16 months. Animals were housed 3–4 per microisolator cage and given commercial food (Purina 5053) and filtered water ad libitum. Animal bedding was Sani chip. The animal room was on a 12 h light/12 h dark cycle. The temperature of the facility was kept at 20 °C–22 °C and relative humidity at 30–70%. 

Animals were monitored at least once a day for any physical or behavioral changes that might indicate distress, discomfort, pain or injury. Mice were weighed at least once a month. If a downward trend in the weight of the mice was noted, they were weighed more frequently, e.g., twice a month or even once a week. The condition was also reported to the animal core facility manager with potential follow-up by the attending veterinarian. 

All animal care facilities used in these studies are accredited by the Association for Assessment and Accreditation of Laboratory Animal Care International (AAALAC). The principles promulgated in the National Research Council’s (NRC) Guide for the Care and Use of Laboratory Animals, Public Law 89-544, and the Animal Welfare Standards were closely followed at all times. All procedures used were approved by the Animal Care and Use Committee at Lawrence Berkeley National Laboratory (LBNL), at Brookhaven National Laboratory (BNL), and at SRI International.

### 2.2. Irradiation Procedures

Animals were exposed to charged particle beams from the NASA Space Radiation Laboratory (NSRL) at BNL [33]. Dosimetric characterizations of the beams, including both depth-dose and dose-uniformity measurements, were conducted by the beam-line physicists. The mice were held briefly unanesthetized individually in plastic boxes (40 mm × 40 mm × 73 mm) drilled with numerous holes to provide abundant airflow. Twelve boxes were assembled in an array one box deep in the 20 cm × 20 cm beam spot with ±2.5% dose uniformity. Animals were irradiated with a single low heavy-ion dose of either 350 MeV/u Oxygen, (LET~20 keV/µm), 260 MeV/u Silicon (LET~70 keV/µm), 1000 MeV/u Titanium (LET~100 keV/µm), or 600 MeV/u Iron ions (LET~193 keV/µm). The dose rate used for each of the beams ranged between 0.2–0.5 Gy/min. A minimum of 5 pulses of radiation were given to ensure uniform distribution of particles for each exposure. Cohorts of animals were also exposed to five fractions of 0.026 or 0.052 Gy of 1000 MeV/u Titanium ions, delivered on five successive days, to simulate chronic exposure. Characteristics of the heavy ion beams used in this paper are presented in Table 1. 

**Table 1:** Beam energies and LETs.

Ion	Energy (MeV/amu)	Entrance LET (keV/μm)
Oxygen	350	20
Silicon	260	70
Titanium	1000	100
Iron	600	193
Cs-137	-	~0.2

During irradiation, animals on the beamline were remotely monitored in the staging area using a video camera system that is mounted in the irradiation cave. Sham-treated control groups of animals were included in each experimental run and the animals were sham-treated by loading them into the clear plastic holders for equivalent exposure times, but they were not irradiated. Since the radiation exposure doses are small, the exposure times were less than 1 min each. After exposure, animals were unloaded to their housing cage with their previous cage mates and transported back to the BLAF within 12–24 h for temporary housing of up to a week until shipment to the LBNL Animal Facility. ^137^Cs γ-rays were used as a low-LET radiation reference. Animals were irradiated locally at SRI International and transported to the animal Facility at LBNL for the 16-month health monitoring. In keeping with historical Harderian Gland tumorigenesis studies, we conducted our lung tumor studies using the same F1 animal strain and study protocol so that we can compare our data to the previously published database. All animals were monitored for 16 months after irradiation prior to scheduled necropsy at SRI International and lung tumor prevalence was only measured at this single time point. No tissues were collected from animals that were moribund prior to the schedule necropsy in this protocol.

### 2.3. Tissue Collection, Sample Processing and Histopathological Analysis

Gross observations of all tissues were conducted during necropsy and recorded. Left lung tissues were fixed for histopathological evaluations and the right lung was snap frozen in liquid nitrogen for further analysis. Fixed tissues were blocked in paraffin, process to slides and hematoxylin/eosin (H/E) stained. The slides of tissue sections were examined by a board-certified veterinarian histopathologist, scored, characterized, and the results were recorded in an electronic data capture system. 

### 2.4. Data Analysis

Lung tumor prevalence is expressed as the number of animals with tumor(s) divided by the total number of animals in the experimental group. Only animals that survived the 16-month period were included in the denominator when calculating prevalence. 

### 2.5. Statistical Analysis

Power calculations were used to determine the appropriate numbers of animals needed in each of the studies to ensure that results obtained are meaningful at the space-relevant low particle radiation doses. Test of statistical significance in the prevalence of lung tumors between radiation and sham groups or between single and fractionated radiation dose groups was tested by Fisher’s exact test. The statistical significance was defined as *p* < 0.05 (two-tails).

## 3. Results

Spontaneous incidences of lung tumors in female CB6F1 arise with age, with tumor prevalence < 5% at ~200 days but reaching levels of up to 40% by 1000 days (33 months of age) [34]. In this study, the reported spontaneous tumor prevalence at ~18 months is ~10%. Table 2 shows the number of animals assigned to the control groups for each study at the start of the study, the number of surviving animals at necropsy and the number of animals with lung tumors. Statistical analyses show that the number of tumors in the sham control groups for the 6 listed cohorts are not significantly different. For this reason, we have pooled all the controls as a single cohort for all the studies presented in this paper.

Of a total of 362 animals at necropsy, 36 animals were found to have lung tumors, resulting in a spontaneous prevalence of 9.94% ± 1.57%. These results are consistent with historical data mentioned above. Histopathological evaluation of the tumor indicated that the majority (~75%) of lung tumors were benign bronchioalveolar adenomas, with ~11% bronchio-alveolar adenocarcinomas. The rest of the tumors were classified as histiocytic sarcomas, hemangiosarcomas or lymphomas. 

**Table 2:** Comparison of tumor prevalence in sham-treated controls.

Beam	Year of Study	Number of Animals at Study Start	At 16-Month Necropsy		
			Number of Animals	Number of Animals with Lung Tumors	% Attrition Prior to Necropsy
Silicon	2011	83	72	2	13.2
Titanium	2012	81	75	10	7.4
Titanium-Fractionation	2013	60	56	4	6.7
Iron	2013	24	23	3	4.2
Cs-137	2013	120	110	15	8.1
Oxygen	2017	30	26	2	13.3
Total		398	362	36	

Lung tumor prevalence for animals that were irradiated with γ-rays is presented in Table 3 below. A single dose of 0.40 Gy, 0.8 Gy or 1.6 Gy resulted in significant increased lung tumors (*p* ≤ 0.01) when compared to the sham-treated controls. However, the lung tumor burden did not appear to further increase with increasing dose after Cs-γ irradiation. Since doses below 0.4 Gy were not used, it is not clear whether the tumor prevalence at doses >0.4 Gy have reached a plateau or whether this is a dose threshold with potential further increases at doses higher than 1.6 Gy. The majority of tumor (between 90–100%) of the tumor were bronchioloalveolar adenomas with an occasional histiocytic sarcoma in the γ-ray-irradiated animals with lung tumors. 

**Table 3:** Prevalence of Lung Tumors after Cs-137 Gamma Irradiation.

Dose (Gy)	Number Animals at Study Start	At 16-Month Necropsy		% Prevalence	*p*-Value *
		Number Animals at Necropsy	Number Animals with Tumors		
sham	398	362	36	9.94 ± 1.57	
0.40	156	148	27	18.2 ± 3.2	0.01
0.80	96	90	20	22.2 ± 4.4	<0.01
1.20	96	77	12	15.6 ± 4.1	0.16
1.60	96	84	18	21.4 ± 4.5	0.01

* *p*-value was obtained by Fisher exact test between respective irradiated dose groups and the sham control group.

We have generated information on LET-dependent changes in both the prevalence and histology of lung tumors arising in the same strain of animals after different particle radiation exposures. Lung tumor prevalence in animals irradiated with Oxygen (O) ions with a LET of ~20 keV/μm (Table 4) increased with increasing dose and appeared to be significantly more tumorigenic than Silicon (Si) (Table 5) or Titanium (Ti) (Table 6). A single low dose of 0.20 Gy of O ions resulted in a significantly higher prevalence compared to 0.16 Gy Si (*p* < 0.05) but not to 0.26 Gy Ti nor 0.20 Gy Fe (*p* > 0.1). A dose-dependent increase in tumor prevalence was observed with no suggestion of a dose threshold above 0.20 Gy. In contrast with the Si and Ti, tumor profile, 50% of the lung tumors after O ion exposures were adenocarcinomas, and 25% were lymphomas in animals in the 0.20 Gy O-ion cohort. Tumors from the higher-dosed groups were predominately adenomas. 

**Table 4:** Prevalence of Lung Tumors after 350 MeV/u Oxygen Irradiation.

Dose (Gy)	Number Animals at Study Start	At 16-Month Necropsy		Prevalence	*p*-Value *
		Number Animals at Necropsy	Number Animals with Tumors		
sham	398	362	36	9.94 ± 1.57	
0.20	60	54	11	20.4 ± 5.5	<0.05
0.40	40	37	8	21.6 ± 6.8	<0.05
0.80	40	33	10	30.3 ± 8.0	<0.01

* *p*-value was obtained by Fisher’s exact test between respective irradiated dose groups and the sham control group.

Low dose irradiated Si-induced lung tumor revealed non-linear dose dependence, suggesting that there may be a dose-threshold of up to 0.16 Gy (Table 5). Tumor burden in animals exposed to <0.16 Gy were similar to background. Tumor prevalence was significantly higher after 0.32 Gy. Greater than 80% of the tumors found were adenoma with ~10% of the tumor found to be lymphomas in the 0.04 Gy and ~10% of the tumor in the 0.08 Gy group classified as histiocytic sarcomas. The lymphomas from these animals were likely due to metastases from other tumor sites such as spleen or thymus in the same animals [35]. 

**Table 5:** Prevalence of Lung Tumors after 260 MeV/u Silicon Irradiation.

Dose (Gy)	Number Animals at Study Start	At 16-Month Necropsy		Prevalence	*p*-Value *
		Number Animals at Necropsy	Number Animals with Tumors		
0	398	362	36	9.94 ± 1.57	
0.04	151	139	12	8.6 ± 2.4	0.74
0.08	132	127	10	7.9 ± 2.4	0.60
0.16	130	101	8	7.9 ± 2.7	0.70
0.32	108	80	15	18.8 ± 4.4	<0.05

* *p*-value was obtained by Fisher’s exact test between respective irradiated dose groups and the sham control group.

Animals exposed to Ti doses as low as 0.03 Gy showed higher, but not- significantly increased lung tumor burden than spontaneous levels after single acute doses (Table 6A). However, no further significant increase in tumor prevalence was observed until the Ti dose increased to >0.30 Gy. Histological evaluations of lung tumors from Ti ions revealed more varied tumor types. Although 90% of the tumors in the 0.03 Gy group were adenomas, ~18% of the tumor were bronchio-adenocarcinomas in animals in the 0.066–0.26 Gy groups. Sporadic lymphomas, histiocytic sarcomas and hemangiosarcomas were also observed. Further increases in carcinomas (~31%), histiocytic sarcomas (6%) were observed in animals irradiated with the highest dose (0.52 Gy) used in these studies. 

**Table 6:** Prevalence of Lung Tumors after 1000 MeV/u Titanium Irradiation.

Dose (Gy)	Number of Animals at Study Start	At 16 Month Necropsy		Prevalence	*p*-Value
		Number of Animals at Necropsy	Number of Animals with Tumors		
sham	398	362	36	9.94 ± 1.57	
A. Single Acute					
0.03	146	135	21	15.6 ± 3.1	0.08 ^a^
0.065	121	107	17	15.9 ± 3.5	0.12 ^a^
0.13	120	110	13	11.8 ± 3.1	0.59 ^a^
0.26	76	69	11	15.9 ± 4.4	0.14 ^a^
0.52	76	62	16	25.8 ± 5.6	< 0.01 ^a^
B. Fractionation					
5 × 0.026 = 0.13	120	104	18	17.3 ± 3.7	0.054 ^a^; 0.33 ^b^
5 × 0.052 = 0.26	120	108	24	22.2 ± 4.0	0.0016 ^a^; 0.34 ^b^

^a^*p*-value was obtained by Fisher’s exact test between respective irradiated dose groups and the sham control group. ^b^ *p*-value was obtained by Fisher’s exact test between fractionated and single acute radiation at the same dose.

In order to simulate the effect of low dose rate in lung tumorigenesis, animals were exposed to 5 daily fractions of 0.026 Gy/day or 0.052 Gy/day of Ti ions for a total dose of 0.13 or 0.26 Gy (Table 6B). These results were compared to the tumor prevalence in animals that were exposed to a single acute exposure (Table 6A). Although results from the fractionation exposure (Table 6B) suggest that the incidences may be slightly higher than that from the acute exposure, statistical comparison between the 2 different dosing regimens show that the values are not statistically significant (*p* > 0.33). Fry et al. [30] exposed mice with the same genetic background to a fractionated regime of 0.025 Gy of fission neutrons over a dose range of 0.05–0.40 Gy and showed that this dosing regimen did not produce more lung adenocarcinomas in single doses of <0.50 Gy, but 0.50 Gy given in two fractions separated by 30 days induced significantly more tumors than a single dose. Our results are consistent with these early low-dose neutron results and further studies with a higher Ti dose may be needed for further confirmation. 

Finally, animals that were exposed to a single dose of 600 MeV/u Iron ions showed that a single low dose of 0.10 Gy or 0.40 Gy of this beam induced a significant increase in lung tumors (Table 7). Interestingly, tumor prevalence at the intermediate 0.20 Gy dose showed a decline in tumor prevalence that is significantly lower than the two neighboring doses. Approximately 90% of the tumors were adenomas in the 0.10 Gy dose group. In the 0.20 Gy dose group, ~80% of the tumors were also adenomas, while 20% were histiocytic sarcomas. Whereas 80% of the tumors in the 0.40 Gy group were adenomas, 10% were adenocarcinomas and 10% were lymphomas. Since the lung is an organ consisting of multiple cell types, it is not clear at this time if this varied distribution of tumor types is due to cell-type specific responses, or due to malignancies in the body that could have impacted the overall lung tumor prevalence. Further evaluations of tumor prevalences with an increased number of animals per dose group and covering a broader dose range may be needed to confirm these findings. 

**Table 7:** Prevalence of Lung Tumors after 600 MeV/u Iron Irradiation.

Dose (Gy)	Number of Animals at Study Start	At 16 Month Necropsy		Prevalence	*p*-Value *
		Number of Animals at Necropsy	Number of Animals with Tumors		
sham	398	362	36	9.94 ± 1.57	
0.10	80	78	19	24.4 ± 4.9	<0.05
0.20	50	47	5	10.6 ± 4.5	0.80
0.40	50	41	12	26.3 ±7.1	<0.01

* *p*-value was obtained by Fisher’s exact test between respective irradiated dose groups and the sham control group.

The percent attrition during the 16-month husbandry period after the various single particle radiation exposures ranged from 4.17% to 13.3%. For most doses, variance analyses showed that animal attritions were not significantly different from the sham-treated controls. However, attrition was higher for the high doses in the Cs, Si and Ti studies and could be attributed to sensitivity of this animal model to long-term injury from the radiation. An analysis of tumor prevalence among the different studies in the sham-treated control groups showed that there was no significant difference (*p*~0.18) suggesting that the variation over a period of >6 yrs is of no concern. 

**Figure 1:**
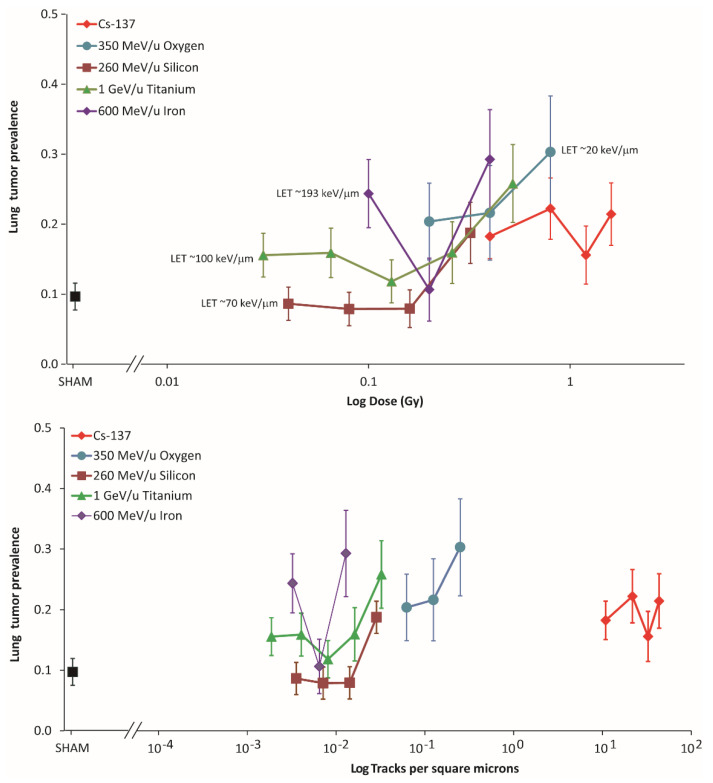
Lung tumor prevalence as a function of dose (**top**) and particle fluence (**bottom**). Black symbol (■) represents the spontaneous lung tumor prevalence in sham-treated animals.

The composite dose and particle fluence results are graphically represented in Figure 1 below. Beam-specific tumor profiles were observed when prevalence was represented as a function of particle fluence. The fact that the tumor prevalence remained similar to sham-treated control values after Si ions at fluences <3 × 10^−3^ particles/μm^2^ suggest that there could be a fluence-dependent threshold for this particle beam. On the other hand, tumor prevalence after similar low fluences of the Ti beam showed significant increased prevalence compared to controls with further increases at fluences >3 × 10^−3^ particles/μm^2^. Interestingly, tumor prevalence after iron ions showed a biphasic response that was not observed in the other ions tested. 

**Figure 2:**
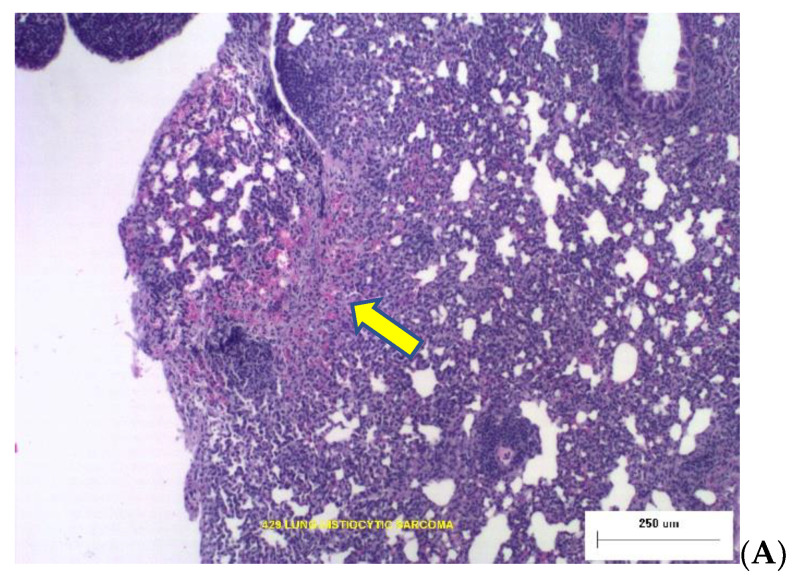

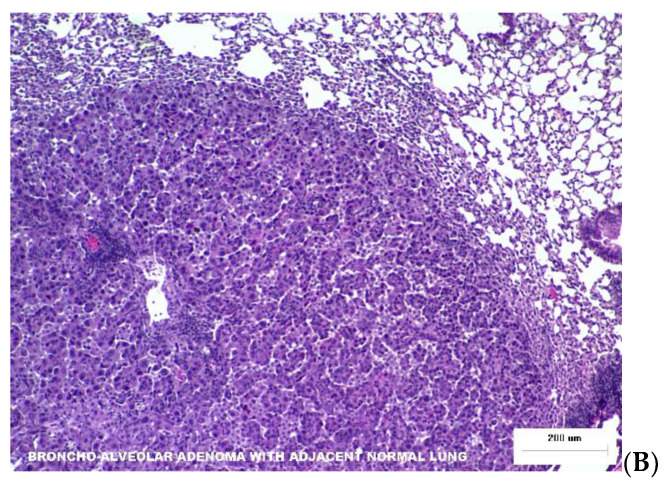
Representative photomicrographs of lung tumor histopathology at necropsy 16 months post irradiation. (**A**) The yellow arrow points to Broncho-Alveolar Adenoma with adjacent normal tissue from an animal that was irradiated with 0.04 Gy Silicon. (**B**) Histiocytic Sarcoma in lung tissues from animal exposed to 0.16 Gy Silicon ions.

Two photomicrographs are included in Figure 2 illustrating the types of dose-dependent lung pathology observed 16-months after exposure to Silicon ions.

## 4. Discussion

We report dose- and LET-dependent prevalence of lung tumorigenesis in CB6F1 mice 16-months after a low single acute exposure for each of four heavy charged particles (oxygen, silicon, titanium or iron) simulating components of the GCR) spectrum using large cohorts of animals to provide statistically meaningful data for space-relevant low doses. Prevalence of HG tumorigenesis in the same animals was previously reported [28] with the exception of the oxygen ion response (manuscript in preparation). In contrast to the low HG spontaneous tumor prevalence from the earlier studies, spontaneous lung tumor prevalence is higher (~10%) and can be considered a limitation in this animal model. Novel lung tumor findings include: (a) Striking non-linear differences in the structure of the low-dose- and low-fluence-dependence of the tumor prevalence responses, and (b) dose- and LET-dependent changes in tumor histologies. We also observed a significant clustering of the lung tumor response for each ion beam compared to what we saw for the prevalence of HG tumors. Lung tumor prevalence after the low-LET reference ^137^Cesium-gamma ray dose response nearly doubled from ~10% at zero dose up to ~18% after 0.4 Gy, but then is rather flat at ~21% at doses up to 1.6 Gy. The question of whether lung tumor prevalence is saturated ≥0.4 Gy is yet to be determined. Lung tumor histology was primarily benign adenomas. In contrast, the tumor prevalence dose response for oxygen ions (at an LET of 20 keV/μm) increased more rapidly from ~22% after 0.2 Gy to ~30% after 0.8 Gy, with a significant increase in diversity of tumor histology, but with the greatest prevalence of adenocarcinomas (50%) at the 0.2 Gy dose.

The lung tumor prevalence for both the silicon (at 70 keV/μm) and titanium (at 100 keV/μm) exposures reveal a relatively flat apparent threshold dose response below 0.2 Gy, with a slight increase in tumors at higher doses. Tumor histologies were primarily adenomas at low dose, but adenocarcinomas and other tumor types appeared at higher doses. Dose fractionation of titanium did not significantly change tumor prevalence. Iron ion tumor prevalence significantly increased with dose but appeared to be bi-phasic. It rose to ~24% at 0.1 Gy and ~27% at 0.4 Gy, but the dose-response at 0.2 Gy was not significantly different than the sham controls. Although this may seem to be surprising, an early study by Fry and colleagues [30] on the percent excess lung adenocarcinomas after a single dose of fission neutrons using the same sex and mouse strain also reported that excess lung adenocarcinomas was lower at 10 cGy than at 5 cGy or 20 cGy. Tumor histologies showed more adenomas at low doses, and more adenocarcinomas and other tumor types at higher doses. Whether this is unique to a high LET value of 190 keV/μm, or the charge of this heavy particle will need to be further explored using intermediate doses between 0.2 to 0.4 Gy or a larger dose range.

Lung tumorigenesis in wild type C57BL/6 mice at ~1.5 years after a single or fractionated 1 Gy of particle radiations with different LETs showed that HZE particles induced not only a higher incidence of lung tumorigenesis than X-rays but also more aggressive tumors and that the relative effectiveness at 1 Gy was >6 [17]. Since space relevant doses for individual particles are not expected to exceed 1 Gy [33,36], the particle radiation doses used in our studies are all below 1 Gy. Despite reports of particle radiation inducing more aggressive tumors [17], the overall number of aggressive tumors appear to be low for each dose/beam combination and no statistical significance was found among the different treatment groups including the controls for this strain of mice and at this time point. Since we only examine tumors at a single 16-month timepoint, it is not clear if the adenomas will progress to carcinomas. 

Weil and colleagues reported an increase in hepatocellular carcinoma (HCC) associated with pulmonary metastasis in C3H mice following 0.2 Gy dose of 300 MeV/u Silicon, compared to 3 Gy dose of γ-rays or unirradiated controls even though the time to appearance of HCC was similar between treatments [12]. Tumors in the HZE-irradiated mice were not more aggressive than those arising from spontaneous or low LET γ-rays or spontaneous [37]. In our studies, lung tumor incidence increased differentially as a function of particle type, LET, dose and particle fluence. Fractionated doses of Titanium ions (5 daily fractions) up to a total dose of <0.30 Gy did not result in increased lung tumor prevalence when compared to the prevalence after a single dose. However, since doses >0.30 Gy were not used in our studies, it is not clear whether higher doses will result in a dramatic increase in tumor prevalence as seen in the fission neutron study. Another limitation of our studies is that we only measured lung tumor prevalence at one time point but not lung tumor incidences over the course of the period. Lung tissues from animals that have died at earlier times were not evaluated and there is, therefore, a possibility that lung tumor prevalence could be underestimated. Further studies to dissect the chronology of lung tumor development in this model is needed to better characterize the natural history of particle-radiation induced lung tumor development. 

The radiation environment in space consists of a mixture of particle beams. Tumorigenesis in animal models after individual beam exposures lays the groundwork for the necessary studies on mixed beams. However, mixed beam studies are nevertheless far more complicated in that, in addition to dose/fluence, the temporal sequence, dose rate and composition of the mixed beams must be taken into consideration. In a recent study, lung cancer tumorigenesis in K-ras LA-1 mice was reported to be dependent on the sequence of H + He + Si vs. Si + He + H [38]. H + He + Si showed increased incidences of premalignant lesions and systemic oxidative stress in mice 100 days post irradiation when compared to Si + He + H or H alone. The results of further analysis of additional experiments with our model system to examine lung tumor prevalence with multi-ion, mixed-GCR beams are in preparation.

In summary, we present lung tumor prevalence data in female CB6F1 mice that indicate both a dose- and LET-dependence for particle beams of different atomic number. Although we see a similar increase in LET-dependence in tumors from the lung as in the HG, there is considerable variability with tumor prevalence clustered more closely together over the same LET range, indicative of tissue-specific responses to particle radiation. In contrast with the HG, which is a pure ‘pocket’ of epithelial cells, multiple cell types with different cell cross-sections constitute the lung tissue, leading to different molecular targets or target sizes. Edmondson et al. [39] reported an overlap in the genetic susceptibility of outbred mice for HZE-ion- and specific γ-ray-induced tumors which they concluded would indicate validity to comparisons with human γ-ray epidemiology for risk assessment. Results from our studies are suggestive that this too can be the case, but further comparative analysis in studies with both sexes will be needed to test the importance of animal breeding (outbred or inbred) and animal strain in susceptibility. Histopathological evaluations of the tumor sections showed that the majority of tumors were benign bronchioloalveolar adenomas with occasional carcinomas. The lymphosarcomas may have resulted from metastases from other sites. Numerous frozen and fixed tissues archived from these experiments involving relatively large animal numbers are available from the NASA Ames Life Sciences Data Archive (ALSDA) tissue repository for NASA’s Space Biology Program (https://www.nasa.gov/ames/research/space-biosciences/alsda) (accessed 31 May 2022) and can be used to further investigate these conclusions.

## Data Availability

All data presented is included in this manuscript.

## References

[1] Barcellos-Hoff M.H., Blakely E.A., Burma S., Fornace A.J., Gerson S., Hlatky L., Kirsch D.G., Luderer U., Shay J., Wang Y. (2015). Concepts and challenges in cancer risk prediction for the space radiation environment. Life Sci. Space Res..

[2] Kreeger P.K., Lauffenburger D.A. (2010). Cancer systems biology: A network modeling perspective. Carcinogenesis.

[3] Chen J., Li Y., Yu T.S., McKay R.M., Burns D.K., Kernie S.G., Parada L.F. (2012). A restricted cell population propagates glioblastoma growth after chemotherapy. Nature.

[4] Driessens G., Beck B., Caauwe A., Simons B.D., Blanpain C. (2012). Defining the mode of tumour growth by clonal analysis. Nature.

[5] Schepers A.G., Snippert H.J., Stange D.E., van den Born M., van Es J.H., van de Wetering M., Clevers H. (2012). Lineage tracing reveals Lgr5+ stem cell activity in mouse intestinal adenomas. Science.

[6] Sullivan J.P., Minna J.D., Shay J.W. (2010). Evidence for self-renewing lung cancer stem cells and their implications in tumor initiation, progression, and targeted therapy. Cancer Metastasis Rev..

[7] Taurin S., Alkhalifa H. (2020). Breast cancers, mammary stem cells, and cancer stem cells, characteristics, and hypotheses. Neoplasia.

[8] Van Es J.H., Sato T., van de Wetering M., Lyubimova A., Yee Nee A.N., Gregorieff A., Sasaki N., Zeinstra L., van den Born M., Korving J. (2012). Dll1^+^ secretory progenitor cells revert to stem cells upon crypt damage. Nat. Cell Biol..

[9] Bielefeldt-Ohmann H., Genik P.C., Fallgren C.M., Ullrich R.L., Weil M.M. (2012). Animal studies of charged particle-induced carcinogenesis. Health Phys..

[10] Shay J.W., Cucinotta F.A., Sulzman F.M., Coleman C.N., Minna J.D. (2011). From mice and men to earth and space: Joint NASA-NCI workshop on lung cancer risk resulting from space and terrestrial radiation. Cancer Res..

[11] Barcellos-Hoff M.H., Mao J.H. (2016). HZE Radiation Non-Targeted Effects on the Microenvironment That Mediate Mammary Carcinogenesis. Front. Oncol..

[12] Weil M.M., Ray F.A., Genik P.C., Yu Y., McCarthy M., Fallgren C.M., Ullrich R.L. (2014). Effects of ^28^Si ions, ^56^Fe ions, and protons on the induction of murine acute myeloid leukemia and hepatocellular carcinoma. PLoS ONE.

[13] Nia A.M., Khanipov K., Barnette B.L., Ullrich R.L., Golovko G., Emmett M.R. (2020). Comparative RNA-Seq transcriptome analyses reveal dynamic time-dependent effects of ^56^Fe, ^16^O, and ^28^Si irradiation on the induction of murine hepatocellular carcinoma. BMC Genom..

[14] Trani D., Nelson S.A., Moon B.H., Swedlow J.J., Williams E.M., Strawn S.J., Appleton P.L., Kallakury B., Nathke I., Fornace A.J. (2014). High-energy particle-induced tumorigenesis throughout the gastrointestinal tract. Radiat. Res..

[15] Delgado O., Batten K.G., Richardson J.A., Xie X.J., Gazdar A.F., Kaisani A.A., Girard L., Behrens C., Suraokar M., Fasciani G. (2014). Radiation-enhanced lung cancer progression in a transgenic mouse model of lung cancer is predictive of outcomes in human lung and breast cancer. Clin. Cancer Res..

[16] Moding E.J., Min H.D., Castle K.D., Ali M., Woodlief L., Williams N., Ma Y., Kim Y., Lee C.L., Kirsch D.G. (2016). An extra copy of p53 suppresses development of spontaneous Kras-driven but not radiation-induced cancer. JCI Insight.

[17] Wang X., Farris Iii A.B., Wang P., Zhang X., Wang H., Wang Y. (2015). Relative effectiveness at 1 gy after acute and fractionated exposures of heavy ions with different linear energy transfer for lung tumorigenesis. Radiat. Res..

[18] McConnell A.M., Konda B., Kirsch D.G., Stripp B.R. (2016). Distal airway epithelial progenitor cells are radiosensitive to High-LET radiation. Sci. Rep..

[19] Cucinotta F.A., Chappell L.J., Kim M.H., Wang M. (2012). Radiation carcinogenesis risk assessments for never-smokers. Health Phys..

[20] Elgart S.R., Little M.P., Chappell L.J., Milder C.M., Shavers M.R., Huff J.L., Patel Z.S. (2018). Radiation Exposure and Mortality from Cardiovascular Disease and Cancer in Early NASA Astronauts. Sci. Rep..

[21] Reynolds R.J., Day S.M. (2018). The effect of competing risks on astronaut and cosmonaut mortality. Life Sci. Space Res..

[22] Reynolds R.J., Day S.M., Nurgalieva Z.Z. (2014). Mortality among Soviet and Russian cosmonauts: 1960–2013. Aviat. Space Environ. Med..

[23] Hassler D.M., Zeitlin C., Wimmer-Schweingruber R.F., Ehresmann B., Rafkin S., Eigenbrode J.L., Brinza D.E., Weigle G., Bottcher S., Bohm E. (2014). Mars’ surface radiation environment measured with the Mars Science Laboratory’s Curiosity rover. Science.

[24] Zeitlin C. (2013). The risky road to Mars--response. Science.

[25] Zeitlin C., Hassler D.M., Cucinotta F.A., Ehresmann B., Wimmer-Schweingruber R.F., Brinza D.E., Kang S., Weigle G., Bottcher S., Bohm E. (2013). Measurements of energetic particle radiation in transit to Mars on the Mars Science Laboratory. Science.

[26] Alpen E.L., Powers-Risius P., Curtis S.B., DeGuzman R. (1993). Tumorigenic potential of high-Z, high-LET charged-particle radiations. Radiat. Res..

[27] Alpen E.L., Powers-Risius P., Curtis S.B., DeGuzman R., Fry R.J. (1994). Fluence-based relative biological effectiveness for charged particle carcinogenesis in mouse Harderian gland. Adv. Space Res..

[28] Chang P.Y., Cucinotta F.A., Bjornstad K.A., Bakke J., Rosen C.J., Du N., Fairchild D.G., Cacao E., Blakely E.A. (2016). Harderian Gland Tumorigenesis: Low-Dose and LET Response. Radiat. Res..

[29] Curtis S.B., Townsend L.W., Wilson J.W., Powers-Risius P., Alpen E.L., Fry R.J. (1992). Fluence-related risk coefficients using the Harderian gland data as an example. Adv. Space Res..

[30] Fry R.J., Powers-Risius P., Alpen E.L., Ainsworth E.J., Ullrich R.L. (1983). High-LET radiation carcinogenesis. Adv. Space Res..

[31] Huang E.G., Wang R.Y., Xie L., Chang P., Yao G., Zhang B., Ham D.W., Lin Y., Blakely E.A., Sachs R.K. (2020). Simulating galactic cosmic ray effects: Synergy modeling of murine tumor prevalence after exposure to two one-ion beams in rapid sequence. Life Sci. Space Res..

[32] Huang E.G., Lin Y., Ebert M., Ham D.W., Zhang C.Y., Sachs R.K. (2019). Synergy theory for murine Harderian gland tumours after irradiation by mixtures of high-energy ionized atomic nuclei. Radiat. Environ. Biophys..

[33] Simonsen L.C., Slaba T.C., Guida P., Rusek A. (2020). NASA’s first ground-based Galactic Cosmic Ray Simulator: Enabling a new era in space radiobiology research. PLoS Biol..

[34] Fry M.R.J., Staffeldt E., Tyler S.A. (1978). Some problems arising in analysis of large scale animal irradiation experiments. Environ. Int..

[35] Zhang Y., Zhang J., Lin C., Wei W., Ren S., Zuo Y. (2012). Overexpression of apoptosis-associated speck-like protein in P388D1 murine lymphoma cells affects metastatic properties. Hematol. Oncol..

[36] Norbury J.W., Slaba T.C., Aghara S., Badavi F.F., Blattnig S.R., Clowdsley M.S., Heilbronn L.H., Lee K., Maung K.M., Mertens C.J. (2019). Advances in space radiation physics and transport at NASA. Life Sci. Space Res..

[37] Udho E.B., Huebner S.M., Albrecht D.M., Matkowskyj K.A., Clipson L., Hedican C.A., Koth R., Snow S.M., Eberhardt E.L., Miller D. (2021). Tumor aggressiveness is independent of radiation quality in murine hepatocellular carcinoma and mammary tumor models. Int. J. Radiat. Biol..

[38] Luitel K., Kim S.B., Barron S., Richardson J.A., Shay J.W. (2020). Lung cancer progression using fast switching multiple ion beam radiation and countermeasure prevention. Life Sci. Space Res..

[39] Edmondson E.F., Gatti D.M., Ray F.A., Garcia E.L., Fallgren C.M., Kamstock D.A., Weil M.M. (2020). Genomic mapping in outbred mice reveals overlap in genetic susceptibility for HZE ion- and gamma-ray-induced tumors. Sci. Adv..

